# Computational Identification of Novel MicroRNAs and Their Targets in *Vigna unguiculata*

**DOI:** 10.1155/2010/128297

**Published:** 2010-08-02

**Authors:** Yongzhong Lu, Xiaoyun Yang

**Affiliations:** ^1^Biology Department, Qingdao University of Science and Technology, Qingdao 266043, China; ^2^Qingdao Academy of Agricultural Sciences, Qingdao 266100, China

## Abstract

MicroRNAs (miRNAs) are a class of endogenous, noncoding, short RNAs directly involved in regulating gene expression at the posttranscriptional level. High conservation of miRNAs in plant provides the foundation for identification of new miRNAs in other plant species through homology alignment. Here, previous known plant miRNAs were BLASTed against the Expressed Sequence Tag (EST) and Genomic Survey Sequence (GSS) databases of *Vigna unguiculata*, and according to a series of filtering criteria, a total of 47 miRNAs belonging to 13 miRNA families were identified, and 30 potential target genes of them were subsequently predicted, most of which seemed to encode transcription factors or enzymes participating in regulation of development, growth, metabolism, and other physiological processes. Overall, our findings lay the foundation for further researches of miRNAs function in *Vigna unguiculata*.

## 1. Introduction

MicroRNAs (miRNAs) are a class of endogenous, small, noncoding, single-stranded RNAs that act as posttranscriptional regulators in eukaryotes [1]. They have been reported to be located mostly within noncoding regions of genomes, and usually transcribed from RNA polymerase II promoters [2, 3]. The generation of mature miRNA is a complicated enzyme-catalyzed process, from the initial transcript pri-miRNA to the precursor (pre-miRNA) with a characteristic hairpin structure, then a miRNA duplex (miRNA : miRNA*) [4]. In the end, it is assembled to the RNA-induced silencing complex (RISC) to direct its activity on a target mRNA, depending on the degree of base-pairing between the miRNA and the responsive element and results in either cleavage or translational repression of the target mRNA. Perfect complementarity generally results in cleavage, such as in plants, whereas imperfect base-pairing leads to translational repression [4, 5]. 

MiRNA genes represent about 1%-2% of the known eukaryotic genomes and constitute an important class of fine-tuning regulators that are involved in several physiological or disease-associated cellular processes [6]. For example, studies in plants have revealed the key roles of miRNAs in diverse regulatory pathways, including growth, development, and defense response against every sort of stress [7–17].

Considering the importance of miRNAs in gene regulation, two major categories of approaches have been applied for miRNA investigation [1]. Compared to the experimental approaches, computation (bioinformatics) methods have been proved to be faster, more affordable, and more effective, contributing mostly to today's plentiful storage in miRBase [1]. Different computational miRNA finding strategies have been developed based on a core principle of looking for conserved sequences between different species that can fold into extended hairpins [18]. The biogenesis of miRNAs suggests that it is possible to find miRNAs by searching Expressed Sequence Tags (ESTs) with known miRNAs. There have been more and more reports about the identification of miRNAs by mining the repository of available ESTs [19–26]. EST analysis makes it possible to rapidly study miRNAs and their functions in species whose genome sequences have not been well known [27].

*Vigna unguiculata* is a vital leguminous crop in tropical and subtropical areas of Asia, Africa, and Latin America, as well as parts of southern Europe and the USA [28]. High protein and carbohydrate contents make it not only important to the human diet, but also suitable as high protein feed and fodder to livestock. With its greater tolerance to heat, drought, and low soil fertility, *Vigna unguiculata* is a particularly valuable component of low-input farming systems of resource-poor farmers. Also, it is able to enhance soil fertility through nitrogen fixation [28, 29]. Until recently limited progress has been made in basic gene discovery and gene regulation in *Vigna unguiculata*, and only two new miRNAs of it have been reported [29, 30]. 

Despite the limited genome resources of* Vigna unguiculata*, publishing of EST and GSS databases in GenBank has provided the chance to get more genetic information. In this study, new miRNAs were mined for the purpose of understanding their roles in regulating growth, development, metabolism, and other physiological processes in *Vigna unguiculata*.

## 2. Materials and Methods

### 2.1. Sequences and Software

The known plant miRNA sequences from *Arabidopsis*, *Brassica*, *Glycine*, *Saccharum*, *Sorghum*, *Vitis*, *Solanum*, *Oryza*, *Triticum*, *Chlamydomonas*, and other plant species were downloaded from the miRNA database miRBase (http://www.mirbase.org/) (Release 14: Sept 2009). After removal of the repeated sequences, 2177 items were left as the reference set. The 187660 EST and 54194 GSS sequences of *Vigna unguiculata* were downloaded from GenBank (http://www.ncbi.nlm.nih.gov/), Blast-2.2.21-ia3 was downloaded from NCBI and set up locally. RNA secondary structure and the free energy were calculated by web server mfold (http://mfold.bioinfo.rpi.edu/) [31]. The software MiRNAassist was applied to improve the analysis efficiency [20].

### 2.2. Prediction of *Vigna unguiculata *MiRNAs

**Figure 1:**
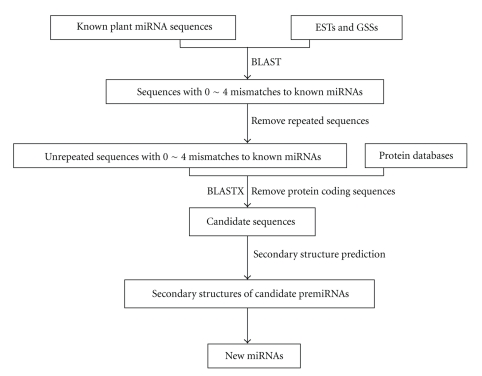
Flowchart of *Vigna unguiculata* miRNA prediction.

The prediction procedure was shown in Figure 1. The sequences of known plant miRNAs were used as query sequences for BLAST searches against the EST and GSS databases, with the BLAST parameters Evalue being 1000 and word-match size between the query and database sequences being 7. Mature miRNA sequences should be no less than 16 nt, and the mismatches should be less than 4. Wherever available, precursor sequences of 400 nt were extracted (200 nt upstream and 200 nt downstream to the BLAST hits) and used for the hairpin structure prediction. If the length of a sequence was less than 400 nt, the entire available sequence was used as a miRNA precursor sequence. These precursor sequences were then BLASTXed online to remove the protein coding sequences (http://blast.ncbi.nlm.nih.gov/Blast.cgi). The retained precursor sequences underwent hairpin structure prediction through web server mfold. Only those meeting the following criteria were designated as miRNA homologs: (1) the RNA sequence folding into an appropriate stem-loop hairpin secondary structure, (2) a mature miRNA sequence located in one arm of the hairpin structure, (3) predicted mature miRNAs with no more than 3 nt substitutions as compared with the known miRNAs, (4) miRNAs having less than 6 mismatches with the opposite miRNA* sequence in the other arm, (5) no loop or break in miRNA* sequences, and (6) predicted secondary structures with higher minimal folding free energy (MFE) and minimal folding free energy index (MFEI), the MEFI usually being over 0.85 [32]. Also, the AU content of pre-miRNA within 30% to 70% was considered [20]. 

### 2.3. Computational Prediction of MiRNA Targets

MiRNA targets prediction was performed by aligning the predicted miRNA sequences with EST sequences of* Vigna unguiculata* through the BLAST program. The targets were screened according to these criteria: the number of mismatches should be less than 4, the minimal free energy of the pairing between miRNA and its target mRNA was lower than −28.2 kcal/mol [33]. After removal of the repeated sequences, the potential target genes were BLASTed against protein databases to predict their function (identity > 25%), since there were no functional annotations for mRNA sequences of *Vigna unguiculata*.

### 2.4. Phylogenetic Analysis of the New MiRNAs

Considering the conservation of miRNAs and their precursors, the precursor sequences of the novel and the known miRNAs in the same family were aligned and phylogenetically analyzed by ClustalW online to investigate their evolutionary relationships (http://www.clustal.org/).

## 3. Results and Discussion

### 3.1. Prediction of *Vigna unguiculata *MiRNAs

**Figure 2:**
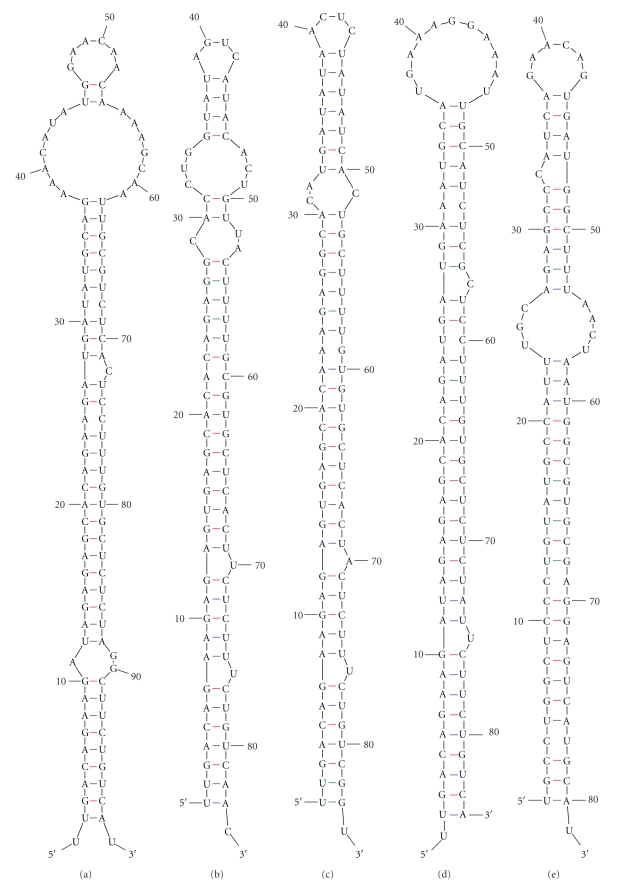

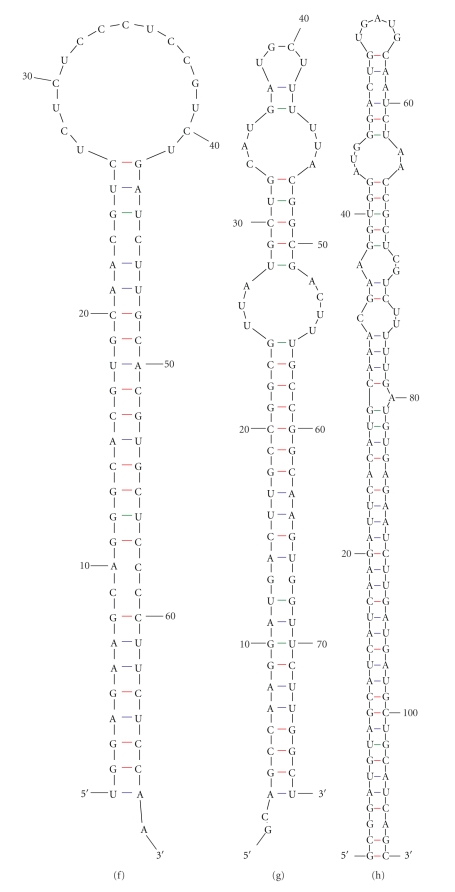

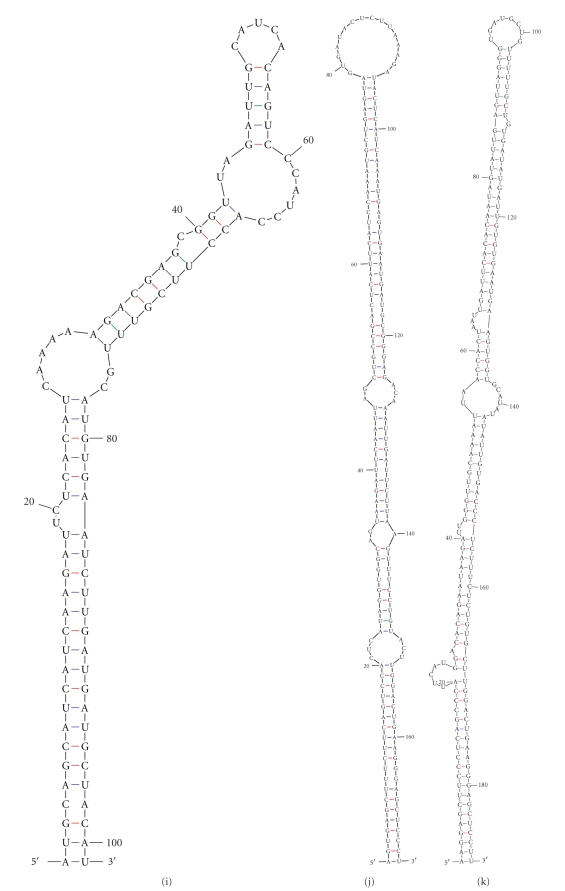

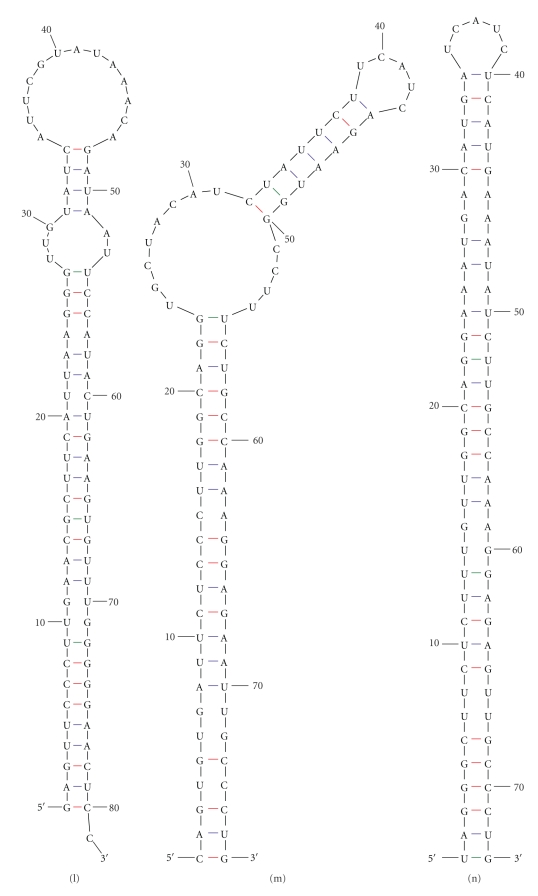

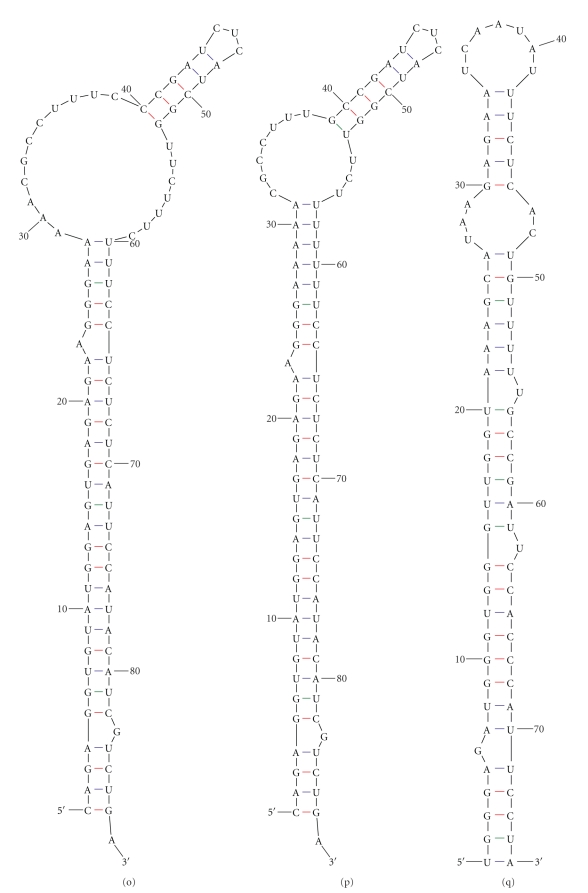

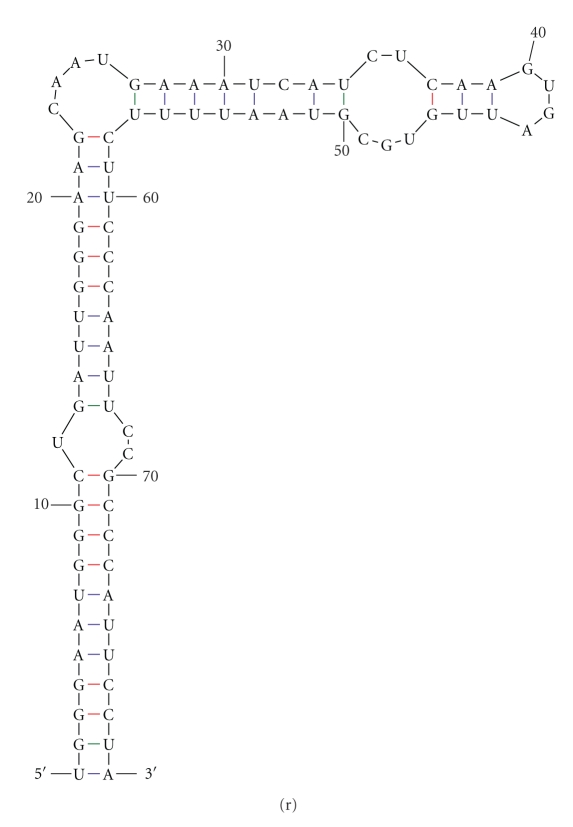
Secondary structures of *Vigna unguiculata* new miRNA precursors. (a) vun-MIR156 precursor 1 (vun-MIR156a (2 to 21); vun-MIR156b (5 to 24); vun-MIR156c (1 to 21); vun-MIR156d (2 to 22)). (b) vun-MIR156 precursor 2 (vun-MIR156f (2 to 22); vun-MIR156g (1 to 21); vun-MIR156h (2 to 21)). (c) vun-MIR156 precursor 3 (vun-MIR156i (2 to 22); vun-MIR156j (1 to 21); vun-MIR156k (2 to 21)). (d) vun-MIR157 precursor (vun-MIR157a (1 to21); vun-MIR157b (2 to 21); vun-MIR157c (2 to 22)). (e) vun-MIR160 precursor (vun-MIR160a (1 to 21); vun-MIR160b (1 to 20)). (f) vun-MIR164 precursor (vun-MIR164 (1 to 21)). (g) vun-MIR169 precursor (vun-MIR169a (2 to 23); vun-MIR169b (2 to 22); vun-MIR169c (1 to 21)). (h) vun-MIR172 precursor 1 (vun-MIR172a (85 to 108); vun-MIR172c (85 to 105); vun-MIR172e (85 to 104)). (i) vun-MIR172 precursor 2 (vun-MIR172b (81 to 101); vun-MIR172d (81 to 100)). (j) vun-MIR319 precursor 1 (vun-MIR319a (152 to 172); vun-MIR319b (151 to 171); vun-MIR319c (152 to 171); vun-MIR319d (151 to 170); vun-MIR319e (152 to 170)). (k) vun-MIR319 precursor 2 (vun-MIR159 (167 to 187); vun-MIR319f (168 to 188); vun-MIR319g (167 to 186); vun-MIR319h (168 to 187); vun-MIR319i (168 to 186)). (l) vun-MIR395 precursor (vun-MIR395a (60 to 81); vun-MIR395b (60 to 80)). (m) vun-MIR399 precursor 1 (vun-MIR399a (57 to 77); vun-MIR399b (57 to 75)). (n) vun-MIR399 precursor 2 (vun-MIR399c (53 to 73); vun-MIR399d (53 to 71)). (o) vun-MIR1507 precursor 1 (vun-MIR1507a (67 to 88); vun-MIR1507b (67 to 87)). (p) vun-MIR1507 precursor 2 (vun-MIR1507c (67 to 88); vun-MIR1507d (67 to 87)). (q) vun-MIR2118 precursor (vun-MIR2118 (54 to 75)). (r) vun-MIR482 precursor (vun-MIR482 (57 to 80)). The position of each miRNA was shown in parentheses.

Sequence and structure homologies are the main theory behind the computer-based approach for miRNAs prediction. As described in Materials and Methods, after BLASTN searches, all blasted hits except coding sequences were maintained for secondary structure analysis, only those in line with the screening criteria were selected as candidates. In the end, 47 potential *Vigna unguiculata* miRNAs belonging to 13 miRNA families were identified, including two known miRNAs (vun-MIR1507a and vun-MIR1507b), and they were named according to Ambros [33]. Information on predicted *Vigna unguiculata* miRNAs, including names, lengths, sources, and other aspects, were listed in Table 1. The length of the 47 predicted miRNAs ranged from 19 nt to 24 nt, while the predicted precursor sequences ranged in length from 68 nt to 188 nt, all forming into typical stem-loop structures, with the mature miRNA either on the 5′ end or the 3′ end (Figure 2). All the MFEIs of these hairpin structures were over 0.85, which was thought to be the gold standard to differentiate miRNAs from other ones [32].

**Figure 3:**
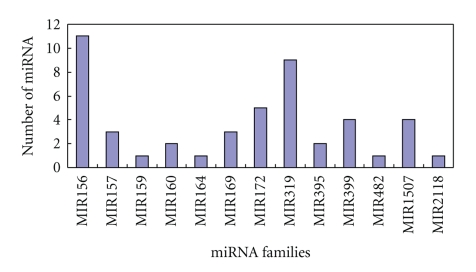
The distribution of new miRNAs in different miRNA families.

*Vigna unguiculata* miRNA precursors were diverse in structure and size, even if they were from the same family, such as those from MIR 156, MIR 172, MIR 319, and MIR 399 families (Figure 2). The distribution of miRNAs in each famiy was different too, some miRNA families have more than 10 members, such as miRNA156 family whereas only one was predicted in other families, such as in miRNA164, miRNA482 and miRNA2118 families (Figure 3). That was consistent with the diversity of miRNAs in other plants [27].

With the availability of sequence resources, computer-based miRNA identification is becoming more and more important than experimental approaches due to its advantages of low cost and high efficiency [1]. At present, three kinds of databases, genome, GSS, and EST, are mainly used for plant miRNA prediction. Considering the unavailability of genome sequences of *Vigna unguiculata*, both EST and GSS databases were searched for miRNA identification. The number and sorts of miRNAs predicted in this work showed that this software-based approach was as feasible and effective as in other work [19–26].

According to Zhang [27], about 10000 ESTs contained one miRNA, so about 20 miRNAs should be predicted theoretically from the total of 241854 ESTs and GSSs in this work. Why as many as 47 were predicted? Generally, plant miRNA clusters have not been frequently observed, so only one plant miRNA can come from the same transcript. In this work, different length of mature miRNAs from the same precursor were regarded as different ones, considering they corresponded to different target genes.

### 3.2. Prediction of *Vigna unguiculata *MiRNA Targets

Based on the complementarity between miRNAs and their target genes in plants, the *Vigna unguiculata* EST database was searched for homology to the new miRNA sequences with a BLASTN algorithm for the discovery of miRNA targets. A total of 30 potential targets for 47 **Vigna unguiculata **miRNAs were identified, and the miRNA target site could be found in 5′UTR, 3′UTR or open reading frame. The miRNAs and their putative targets with known functions were listed in Table 2. These potential miRNA targets belonged to a number of gene families that had different biological functions. 

Many of these targets were transcription factors that controlled plant development and phase change from vegetative growth to reproductive growth. Several studies indicated that MIR156/157 targeted squamosa promoter binding protein (SBP)-like (SPL) genes, a plant-specific family of transcription factors involved in early flower development and vegetative phase changes [34, 35]. Auxin response factors (ARF), a plant-specific family of DNA binding proteins, were involved in hormone signal transduction [36]. AP(2)APETALA2 transcription factors were involved in several developmental processes in *Arabidopsis thaliana* embryo, endosperm, and seed coat development [37]. TCP family transcription factors have been reported to play roles in various aspects of plant development [38, 39]. Heat shock transcription factor was reported to participate in heat shock response to environmental stress [40].

In addition to the transcription factors, another important part of the predicted targets were various kinds of enzymes such as trehalose-6-phosphate synthase, taxadien-5-alpha-ol O-acetyltransferase, delta-aminolevulinic acid dehydratase, porphobilinogen synthase, and alpha/beta hydrolase, which might play roles in various metabolic pathways [41–44].

Identification of genes targeted by miRNAs is widely believed to be an important step toward understanding the role of miRNAs in gene regulatory networks. EST database searches play a vital role for the discovery of miRNA targets in plants based on the homology between miRNA and its target sequences [45]. The protein databases applied to predict the functions of the target genes in this work included nonredundant protein sequences, reference protein sequences and so on. Though this method may be error-prone, it provides a fast way to know something about the gene function.

Our prediction of target genes for the 47 miRNAs also revealed that not just one gene might be regulated by individual miRNA (Table 2). This result was consistent with recent findings in other plant species. Wu et al. also reported that multiple miRNAs could target the same gene. All these suggested that miRNA research should focus on networks more than on individual connections between miRNA and strongly predicted targets [46].

### 3.3. Phylogenetic Analysis of the New MiRNAs

Plant miRNAs are highly conserved among distantly related plant species, both in terms of primary and mature miRNAs [27]. Comparison of the precursor sequences of the predicted miRNAs with other members in the same family showed that most members could be found to have a high degree of sequence similarity with others, for example, the precursor sequence similarity between vun-MIR156 and other MIR156 members was over 60%, that between vun-MIR157 and ath-MIR157a, gra-MIR157a, bol-MIR157a, or sly-MIR157b was over 80% (data not shown).

**Figure 4:**
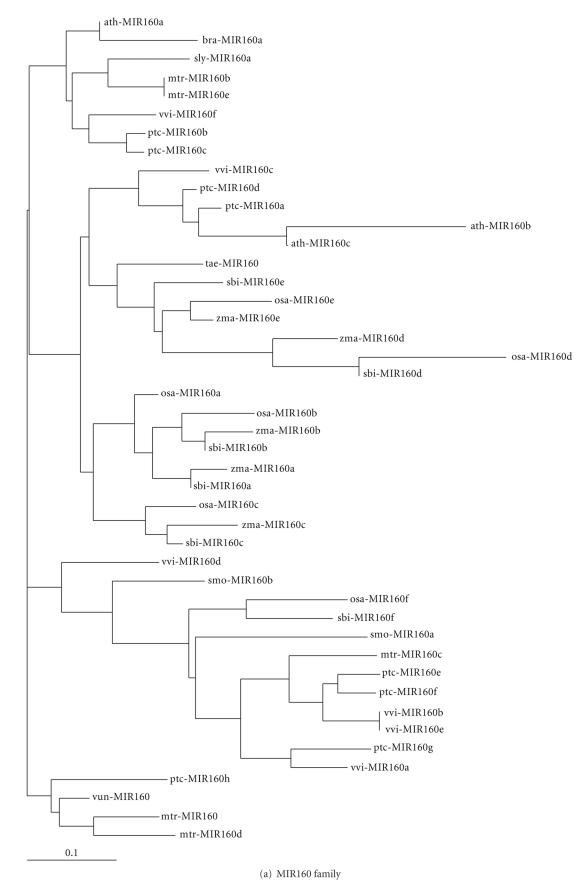

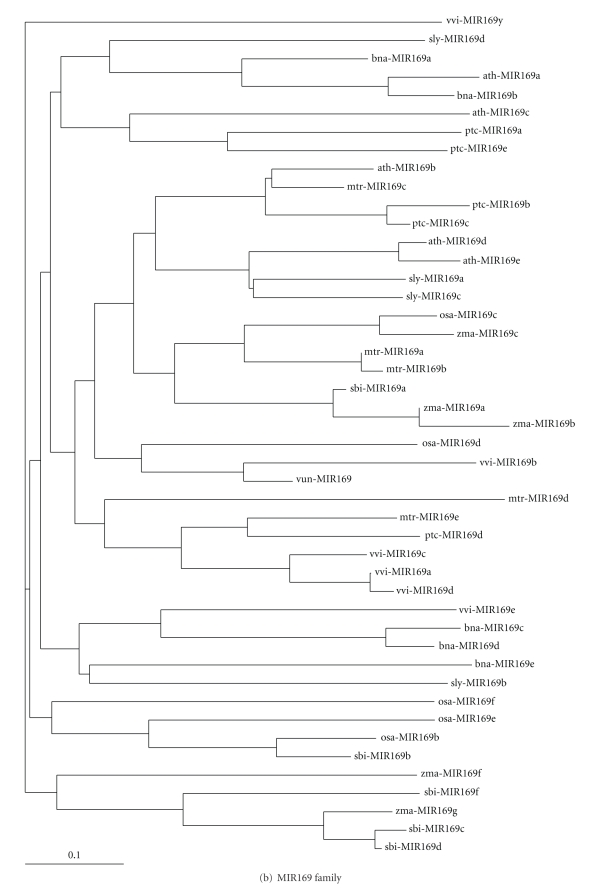

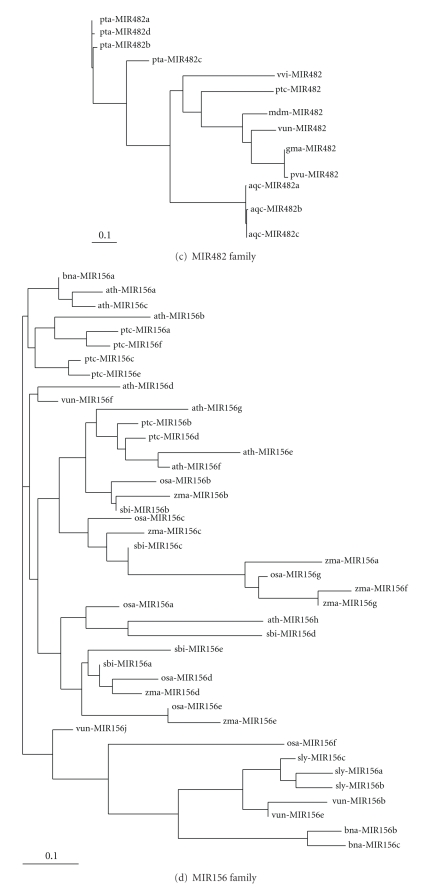

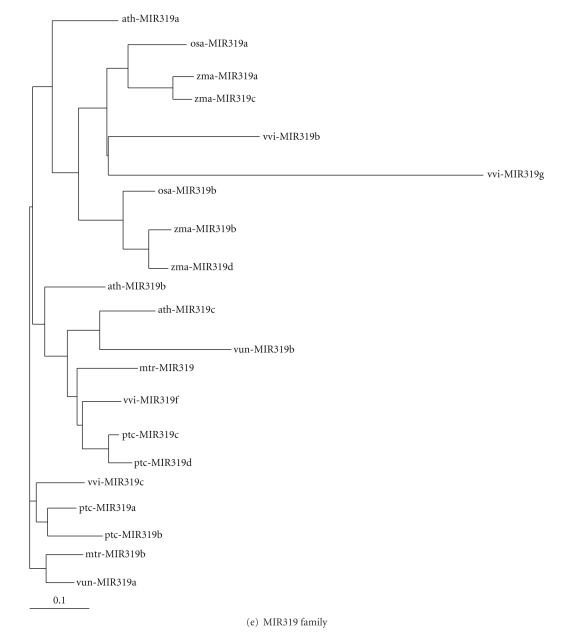
Phylogenetic analysis of miRNAs in different families.

The conservation of mature miRNAs and premiRNAs provides the chance to investigate their evolutionary relationships. Based on the premiRNA sequence comparisons, the evolutionary relationships of *Vigna unguiculata* miRNAs with other members from the same families were analyzed using the ClustalW online. It could be seen from the phylogenetic trees that in different families, the evolutionary relationships of *Vigna unguiculata* miRNAs with other species were different; for example, in MIR160 family, vun-MIR160 and mtr-MIR160 were on the same branch, while in MIR169 family, vun-MIR169 had close relationship with wi-MIR169, and in MIR482 family, vun-MIR482 was closely related to pvu-MIR482 and gma-MIR482 (Figures 4(a)–4(c)). Also, it could be seen that different *Vigna unguiculata* miRNA members in the same family were often distantly related, such as in MIR156 and MIR319 families (Figures 4(d) and 4(e)). These results suggested that different miRNAs might evolve at different rates not only within the same plant species, but also in different ones.

## 4. Conclusions

In this paper, with a bioinformatic method, 47 miRNAs were identified from the EST and GSS databases of* Vigna unguiculata*; also about 30 potential targets of them were predicted, which appeared to be related to the development, growth, metabolism, and other physiological processes such as stress response of* Vigna unguiculata*. The findings from this study will contribute to further researches of miRNAs function and regulatory mechanisms in *Vigna unguiculata*.

## Figures and Tables

**Table 1:** New miRNAs identified in *Vigna unguiculata. *

New miRNA	Gene ID	Source	miRNA sequence (5′3′)	NM/nt	LM/nt	LP/nt	Location	A + U (%)	MFEI
vun-MIR156a	146512093	GSS	UGACAGAAGAUAGAGAGCAC	0	20	100	5	0.58	1.06
vun-MIR156b	146512093	GSS	CAGAAGAUAGAGAGCACAGA	2	20	92	5	0.58	1.06
vun-MIR156c	146512093	GSS	UUGACAGAAGAUAGAGAGCAC	0	21	100	5	0.59	1.06
vun-MIR156d	146512093	GSS	UGACAGAAGAUAGAGAGCACA	1	21	100	5	0.58	1.06
vun-MIR156e	190469749	EST	CAGAAGAUAGAGAGCACAGA	2	20	77	5	0.58	1.18
vun-MIR156f	190510876	EST	UGACAGAAGAGAGUGAGCACA	0	21	82	5	.055	1.09
vun-MIR156g	190510876	EST	UUGACAGAAGAGAGUGAGCAC	3	21	84	5	0.55	1.07
vun-MIR156h	190510876	EST	UGACAGAAGAGAGUGAGCAC	0	20	82	5	0.55	1.09
vun-MIR156i	190416464	EST	UGACAGAAGAGAGUGAGCACA	0	21	82	5	0.57	1.29
vun-MIR156j	190416464	EST	UUGACAGAAGAGAGUGAGCAC	3	21	84	5	0.58	1.30
vun-MIR156k	190416464	EST	UGACAGAAGAGAGUGAGCAC	0	20	82	5	0.57	1.29
vun-MIR157a	190469749	EST	UUGACAGAAGAUAGAGAGCAC	1	21	84	5	0.60	1.32
vun-MIR157b	190469749	EST	UGACAGAAGAUAGAGAGCAC	0	20	84	5	0.60	1.31
vun-MIR157c	190469749	EST	UGACAGAAGAUAGAGAGCACA	1	21	84	5	0.60	1.31
vun-MIR159	182406416	EST	CUUGGACUGAAGGGAGCUCCU	1	21	186	3	0.60	1.00
vun-MIR160a	190509770	EST	UGCCUGGCUCCCUGUAUGCCA	0	21	81	5	0.48	1.05
vun-MIR160b	190509770	EST	UGCCUGGCUCCCUGUAUGCC	0	20	81	5	0.48	1.05
vun-MIR164	146508076	GSS	UGGAGAAGCAGGGCACGUGCA	0	21	68	5	0.41	0.90
vun-MIR169a	190499818	EST	CAGCCAAGGAUGACUUGCCGGC	2	22	76	5	0.46	1.04
vun-MIR169b	190499818	EST	CAGCCAAGGAUGACUUGCCGG	0	21	76	5	0.46	1.04
vun-MIR169c	190499818	EST	GCAGCCAAGGAUGACUUGCCG	1	21	77	5	0.46	1.02
vun-MIR172a	190415307	EST	AGAAUCUUGAUGAUGCUGCAUCAG	2	24	109	3	0.55	1.02
vun-MIR172b	190415307	EST	UGAAUCUUGAUGAUGCUACAU	0	21	101	3	0. 57	0.89
vun-MIR172c	190415307	EST	AGAAUCUUGAUGAUGCUGCAU	0	21	101	3	0.57	1.03
vun-MIR172d	190415307	EST	UGAAUCUUGAUGAUGCUACA	2	20	100	3	0.57	0.87
vun-MIR172e	190415307	EST	AGAAUCUUGAUGAUGCUGCA	1	20	101	3	0.56	0.99
vun-MIR319a	182650666	EST	UUGGACUGAAGGGAGCUCCCU	0	21	172	3	0.58	0.99
vun-MIR319b	182650666	EST	CUUGGACUGAAGGGAGCUCCC	0	21	174	3	0.59	1.03
vun-MIR319c	182650666	EST	UUGGACUGAAGGGAGCUCCC	0	20	174	3	0.59	1.03
vun-MIR319d	182650666	EST	CUUGGACUGAAGGGAGCUCC	0	20	167	3	0.58	1.01
vun-MIR319e	182650666	EST	UUGGACUGAAGGGAGCUCC	0	19	167	3	0.58	1.01
vun-MIR319f	182406416	EST	UUGGACUGAAGGGAGCUCCUU	0	21	188	3	0.59	1.00
vun-MIR319g	182406416	EST	CUUGGACUGAAGGGAGCUCC	0	20	184	3	0.58	1.00
vun-MIR319h	182406416	EST	UUGGACUGAAGGGAGCUCCU	0	20	186	3	0.59	1.00
vun-MIR319i	182406416	EST	UUGGACUGAAGGGAGCUCC	0	19	184	3	0.58	0.98
vun-MIR395a	146525281	GSS	CUGAAGUGUUUGGGGGAACUCC	0	22	81	3	0.59	1.04
vun-MIR395b	146525281	GSS	CUGAAGUGUUUGGGGGAACUC	0	21	80	3	0.60	1.04
vun-MIR399a	182643512	EST	UGCCAAAGGAGAAUUGCCCUG	0	21	77	3	0.52	0.91
vun-MIR399b	182643512	EST	UGCCAAAGGAGAAUUGCCC	0	19	77	3	0.52	0.91
vun-MIR399c	182648023	EST	UGCCAAAGGAGAGUUGCCCUG	0	21	73	3	0.56	1.16
vun-MIR399d	182648023	EST	UGCCAAAGGAGAGUUGCCC	1	19	69	3	0.55	1.11
vun-MIR482	146504713	GSS	UCUUCCCAAUUCCGCCCAUUCCUA	0	24	80	3	0.56	0.96
vun-MIR1507a	182400468	EST	UCUCAUUCCAUACAUCGUCUGA	0	22	88	3	0.53	1.03
vun-MIR1507b	182400468	EST	UCUCAUUCCAUACAUCGUCUG	0	21	87	3	0.53	1.03
vun-MIR1507c	190551234	EST	UCUCAUUCCAUACAUCGUCUGA	0	22	88	3	0.55	1.18
vun-MIR1507d	190551234	EST	UCUCAUUCCAUACAUCGUCUG	0	21	87	3	0.54	1.16
vun-MIR2118	190540631	EST	UUGCCGAUUCCACCCAUUCCUA	1	22	75	3	0.57	1.11

NM: Number of mismatch; LM: Length of mature miRNA; LP: Length of precursor; MFEI: Minimal folding free energy index.

**Table 2:** The potential targets of newly identified miRNAs in *Vigna unguiculata. *

miRNA	Targeted gene	Targeted protein	Possible function	
MIR156c/MIR157a	190524055(1)	squamosa promoter-binding protein	transcription factor	3′UTR
	190515614(0)	squamosa promoter-binding protein	transcription factor	ORF
	190473822(0)	squamosa promoter-binding protein	transcription factor	5′UTR
	190427748(1)	LIGULELESS1 protein	transcription factor	ORF
MIR156j//MIR156g	190416464(0)	rho-related protein racG	signal transduction	5′UTR
MIR160b	190447817(0)	auxin response factor	stress response	ORF
MIR164	182656787(2)	NAC domain protein	transcription factor	ORF
	182647558(3)	NAC domain protein	transcription factor	ORF
MIR172b	182399040(3)	protein kinase family protein	metabolism	ORF
	190449132(3)	trehalose-6-phosphate synthase	metabolism	ORF
MIR172c	182652950(2)	transcription factor AHAP2	transcription factor	ORF
	182404391(2)	AP2 domain-containing transcription factor	transcription factor	ORF
MIR172e	190524349(3)	alpha/beta hydrolase	metabolism	ORF
	190501430(2)	transcription factor APETALA2	transcription factor	ORF
	182389603(2)	protein AINTEGUMENTA, putative	transcription factor	ORF
MIR319e/MIR319i	190555544(3)	taxadien-5-alpha-ol O-acetyltransferase	metabolism	ORF
182406172(n)	182402586(3)	ATP synthase alpha chain, mitochondrial	metabolism	3′UTR
MIR319h	190541283(3)	TCP family transcription factor	transcription factor	ORF
MIR399b	190527737(3)	delta-aminolevulinic acid dehydratase	metabolism	ORF
	190504725(3)	50S ribosomal protein L22,	metabolism	ORF
	190478794(2)	arginine biosynthesis protein argJ 1ornithine acetyltransferase (OAT) family	metabolism	ORF
	190452752(1)	cytochrome	metabolism	5′UTR
	182643288(3)	aldehyde dehydrogenase	metabolism	ORF
MIR2118	190540632(0)	cytoplasmic tRNA adenylyltransferase 1chromatin assembly factor 1 subunit p90 ATP-dependent RNA helicase DBP3	translation	ORF

Parentheses indicate the number of mismatches between the miRNA and mRNA; ORF: open reading frame; UTR: untranslated region.
